# On evidence fiascos and judgments in COVID-19 policy

**DOI:** 10.1007/s40656-021-00410-w

**Published:** 2021-04-16

**Authors:** Saana Jukola, Stefano Canali

**Affiliations:** 1 Institute for Medical Humanities, Universitätsklinikum Bonn, Bonn, Germany; 2 Department of Electronics, Information and Bioengineering, Politecnico Di Milano, Milan, Italy

**Keywords:** Evidence-based medicine, COVID-19 pandemic, Philosophy of medicine

## Abstract

Calls for evidence-based approaches to COVID-19 have sparked up discussions on the use of evidence for policy. In this note, we expand these discussions: while the debate has mostly focused on the types of evidence to be used for policy, we argue that the assessment of judgments involved in data practices and evidence production should play a central role in evaluating policy.

In this note, we analyse the use of data and evidence as a basis of mitigation measures of the COVID-19 pandemic.^1^ We first briefly review the scientific and philosophical debate on COVID-19 evidence and then expand it to shift the focus to judgements. Our worry is that these discussions on mitigation measures have focused on the question of which types of data and evidence pandemic policies should be based on, while the role of judgments in evidence and data practices has been underappreciated.

Throughout the COVID-19 pandemic, governments around the world have relied on teams of scientific experts to guide their policy-making. Many commentators have criticised these efforts by arguing that COVID-19 policies have a significant flaw: they are not truly evidence-based. A prominent representative of these critiques has been epidemiologist and meta-scientist John P.A. Ioannidis, who claimed that the quality and reliability of COVID-19 research are very low. Ioannidis has criticised many interventions, especially lockdown measures, arguing that pandemic policy runs the risk of becoming a “once-in-a-century evidence fiasco” (Ioannidis, 2020). Philosophers of science and medicine such as Johnathan Fuller soon entered this debate. They highlighted that the criticism of COVID-19 policies voiced by Ioannidis and others reflects the values and principles of Evidence-Based Medicine (EBM), an approach to biomedical research and practice that is based on a specific and often contested ranking of different types of evidence (Fuller, 2020).

The criterion according to which different study types are evaluated in EBM is internal validity. Meta-analyses and randomized controlled trials (RCTs) are placed on the top of the evidence hierarchy because they are taken to have greater internal validity. These studies control various biases using randomisation and blinding (in the case of RCTs) and strict pre-established guidelines for conducting the analysis (in the case of meta-analyses). Correspondingly, expert opinion and other types of evidence, for instance from observational studies, are considered to be unreliable because they cannot control possible biasing effects of subjective preferences and confounding. These assumptions about evidence types and assessment explain why many COVID-19 mitigation measures have been criticised as lacking in evidence and quality: because they are often grounded on observational studies or expert opinion.

However, evaluating COVID-19 measures on the basis of EBM can be criticized for reasons that have been raised in previous literature critical of EBM. For example, the feasibility of high-quality RCTs on the effectiveness of mitigation measures is questionable. Perhaps more important is the problem of extrapolability and generalizability. This problem normally affects RCTs, but is particularly grave in the COVID-19 context, where outcomes of an intervention conducted in one social and cultural context need to be applied in very different contexts (Broadbent & Smart, 2020).

We think that this debate on evidence is crucial for COVID-19 policy, but so far it has mostly focused on the *types* of evidence used in policy. We want to introduce an additional line of criticism of evidence-based policy which is particularly significant in this case. At its core, EBM has very specific ideal, namely the exclusion of individual, theory- and value-laden judgments from the research process (Jukola, 2017; Rocca, 2018). The strict rules of conducting RCTs and meta-analyses are precisely meant to constrain the judgments and preferences of individuals involved in the process of producing evidence, thus supposedly enabling objective and independent inferences (Stegenga, 2018). However, even formal rules do not determine how research should be carried out in concrete situations. This means that different theory- and value-laden judgments (considering for example the nature of the phenomena under investigation) are required (Jukola, 2017; Rocca, 2018).

This observation about the role of judgements is central with respect to COVID-19 data. Contrary to the EBM view of evidence as a neutral and value-free entity, judgments and assumptions play a crucial role in determining what the data are evidence for (Canali, 2020). For instance, viewing sewage data as a representation of COVID-19 outbreaks involves judgements at the level of sampling, the comparability of different geographical areas and the theoretical connections between sewage and infection. Similarly, determining the number of deaths from COVID-19 is far from a simple counting procedure, since  COVID-19 deaths usually happen in the presence of many pre-existing conditions (Amoretti & Lalumera, 2021). As a consequence, judgments made in data practices are necessary and influence what can be used as evidence of death. This in turn will have consequences on rates such as case fatality that are crucial for pandemic research and policy.

Given the central role that diverse judgments play in the production and use of evidence, we argue that they should be at the centre of transparent discussions and scrutiny. Debating the types of evidence that are used as a basis for policy is important. But it is not enough and leaves the role of judgments unquestioned. In the current pandemic, we have unfortunately witnessed severe shortcomings and failures of data strategies due to the lack of reliable information on the different types of tests, collection and judgements (Leonelli, 2021). While the prominence of some epidemiological models has led to increasing scrutiny and reflections on the epistemic limits of modelling, more should be done to present and coordinate the role of judgments that influence these models and data practices. As shown by philosophers and data studies scholars Sabina Leonelli and Niccolò Tempini (2018), this is not only important for policy: documenting judgments, assumptions and values enhances the effective integration of epidemiological data and the quality of research. As a way of preparing for future biomedical emergencies, we should expand this approach to judgments and see them as key contextual elements of both evidential reasoning and policy-making.

## Footnotes

1 This note belongs to the Topical Collection “Seeing Clearly Through COVID-19: Current and future questions for the history and philosophy of the life sciences”, edited by G. Boniolo and L. Onaga.

## References

[CR1] Amoretti MC, Lalumera E (2021). COVID-19 as the underlying cause of death: Disentangling facts and values. History and Philosophy of the Life Sciences.

[CR2] Broadbent, A. & Smart, B. (2020). Why a one-size-fits-all approach to COVID19 could have lethal consequences. *The Conversation*. https://theconversation.com/why-aone-size-fits-all-approach-to-covid-19-could-have-lethal-consequences-134252 (Accessed March 2021).

[CR3] Canali S (2020). Making evidential claims in epidemiology: Three strategies for the study of the exposome. Studies in History and Philosophy of Biological and Biomedical Sciences.

[CR4] Fuller, J. (2020). Models v. Evidence. *Boston Review*. Available at: https://bostonreview.net/science-nature/jonathan-fuller-models-v-evidence (Accessed March 2021).

[CR5] Ioannidis, J.P.A. (2020). A fiasco in the making? As the coronavirus pandemic takes hold, we are making decisions without reliable data. *STAT*. Available at: https://www.statnews.com/2020/03/17/a-fiasco-in-the-making-as-the-coronavirus-pandemic-takes-hold-we-are-making-decisions-without-reliable-data/ (Accessed March 2021).

[CR6] Jukola S (2017). On ideals of objectivity, judgments, and bias in medical research—A comment on Stegenga. Studies in History and Philosophy of Biological and Biomedical Sciences.

[CR7] Leonelli, S. (2021). Data science in times of pan(dem)ic. *Harvard Data Science Review,**3*(1).

[CR8] Leonelli, S., & Tempini, N. (2018). Where health and environment meet: the use of invariant parameters in big data analysis. *Synthese*. 10.1007/s11229-018-1844-2 PMC8550214 34720225

[CR9] Rocca E (2018). The judgments that evidence-based medicine adopts. Journal of Evaluation in Clinical Practice.

[CR10] Stegenga, J. (2018). *Medical nihilism*. Oxford University Press.

